# Real-time adaptive planning method for radiotherapy treatment delivery for prostate cancer patients, based on a library of plans accounting for possible anatomy configuration changes

**DOI:** 10.1371/journal.pone.0213002

**Published:** 2019-02-28

**Authors:** Maria Antico, Peter Prinsen, Francesco Cellini, Alice Fracassi, Alfonso A. Isola, David Cobben, Davide Fontanarosa

**Affiliations:** 1 School of Chemistry, Physics and Mechanical Engineering, Queensland University of Technology, Brisbane, Queensland, Australia; 2 Institute of Health & Biomedical Innovation, Queensland University of Technology, Brisbane, QLD, Australia; 3 Delft University of Technology, Delft, The Netherlands; 4 Philips Research, Oncology Solutions Department, Eindhoven, The Netherlands; 5 UOC Radioterapia Oncologica, Dipartimento di Diagnostica per Immagini, Radioterapia Oncologica ed Ematologia, Istituto di Radiologia, Fondazione Policlinico A. Gemelli, IRCCS—Università Cattolica Sacro Cuore, Roma, Italia; 6 University of Rome Tor Vergata, Rome, Italy; 7 North West Cancer Centre, Altnagelvin Hospital, Derry-Londonderry, Northern Ireland; 8 The University of Manchester, Division of Cancer Studies, School of Medical Sciences, Faculty of Biology, Medicine and Health, Manchester, United Kingdom; 9 The Christie NHS Foundation Trust, Clinical Oncology, Manchester, United Kingdom; 10 School of Clinical Sciences, Queensland University of Technology, Gardens Point Campus, Brisbane, QLD, Australia; Northwestern University Feinberg School of Medicine, UNITED STATES

## Abstract

**Background and purpose:**

In prostate cancer treatment with external beam radiation therapy (EBRT), prostate motion and internal changes in tissue distribution can lead to a decrease in plan quality. In most currently used planning methods, the uncertainties due to prostate motion are compensated by irradiating a larger treatment volume. However, this could cause underdosage of the treatment volume and overdosage of the organs at risk (OARs). To reduce this problem, in this proof of principle study we developed and evaluated a novel adaptive planning method. The strategy proposed corrects the dose delivered by each beam according to the actual position of the target in order to produce a final dose distribution dosimetrically as similar as possible to the prescribed one.

**Material and methods:**

Our adaptive planning method was tested on a phantom case and on a clinical case. For the first, a pilot study was performed on an in-silico pelvic phantom. A “library” of intensity modulated RT (IMRT) plans corresponding to possible positions of the prostate during a treatment fraction was generated at planning stage. Then a 3D random walk model was used to simulate possible displacements of the prostate during the treatment fraction. At treatment stage, at the end of each beam, based on the current position of the target, the beam from the library of plans, which could reproduce the best approximation of the prescribed dose distribution, was selected and delivered. In the clinical case, the same approach was used on two prostate cancer patients: for the first a tissue deformation was simulated in-silico and for the second a cone beam CT (CBCT) taken during the treatment was used to simulate an intra-fraction change. Then, dosimetric comparisons with the standard treatment plan and, for the second patient, also with an isocenter shift correction, were performed.

**Results:**

For the phantom case, the plan generated using the adaptive planning method was able to meet all the dosimetric requirements and to correct for a misdosage of 13% of the dose prescription on the prostate. For the first clinical case, the standard planning method caused underdosage of the seminal vesicles, respectively by 5% and 4% of the prescribed dose, when the position changes for the target were correctly taken into account. The proposed adaptive planning method corrected any possible missed target coverage, reducing at the same time the dose on the OARs. For the second clinical case, both with the standard planning strategy and with the isocenter shift correction target coverage was significantly worsened (in particular uniformity) and some organs exceeded some toxicity objectives. While with our approach, the most uniform coverage for the target was produced and systematically the lowest toxicity values for the organs at risk were achieved.

**Conclusions:**

In our proof of principle study, the adaptive planning method performed better than the standard planning and the isocenter shift methods for prostate EBRT. It improved the coverage of the treatment volumes and lowered the dose to the OARs. This planning method is particularly promising for hypofractionated IMRT treatments in which a higher precision and control on dose deposition are needed. Further studies will be performed to test more extensively the proposed adaptive planning method and to evaluate it at a full clinical level.

## Introduction

One of the clinically most wide-spread treatment techniques in external beam radiation therapy (EBRT) for prostate cancer is intensity-modulated radiation therapy (IMRT), where the intensities of the treatment beams are optimized with an inverse planning method based on specific treatment objectives[1][2]. Standard IMRT treatments, with techniques like step-and-shoot or sliding windows leaf sequencing algorithms, can be rather long (20 minutes or more). Therefore, it is important to ensure that the position of the treatment volume, which may move during treatment, is properly monitored in order to detect possible misdosage.

In prostate cancer radiotherapy (RT) treatments, the treatment volume is typically the whole prostate gland with or without the seminal vesicles[3]. The prostate gland is a relatively mobile organ. During a treatment fraction, due to several physiologic factors including for example bladder filling, peristaltic and respiratory motion[4][5], there is evidence that the prostate might drift more than 1 cm from its original position[6][7][8][9]. To mitigate the effects of this motion and to compensate for other treatment setup uncertainties (e.g. errors in contour delineation) usually a margin is added to the treatment volume to define the planning target volume (PTV) and the whole volume is irradiated [10]. However, due to the significant inter- and intra-patient intra-fraction displacement variability [7][8][9] and the increasing probability of prostate large shifts over time[6][7][8][11], imposing a standard static margin might result in incorrect dose deposition[6][7][8][11][12][13].

A possible approach to reduce the negative effects of large margins, such as higher dose to organs at risk for example, is to monitor the position of the treatment volumes prior to and/or during every treatment fraction, a process usually referred to as image-guided radiation therapy (IGRT) (e.g. [14][15]). The information on the position can then be used to adapt the treatment based on the current position of the target (ART, adaptive RT[16]). For example, performing a realignment of the patient before each beam[17], in particular when the treatment volume is outside of some specific thresholds[18], or recalculating the dose off-line after the end of each fraction and adapting the dose distributions in the following fractions to compensate for possible errors[19]. Not many examples of adaptation in real-time are available in literature, due to the complexity of this approach for inverse planning approaches. The most relevant works include using a robotic couch to rigidly shift the patient either to compensate for respiration[20] or to re-align the patient in case of prostate motion[21]. However, the first solution does not correct for organs induced prostate motion, which is the main cause of prostate drifts[5][22].The second solution does not take into account the deformations of the normal tissue surrounding the prostate and the possible relative motion of the seminal vesicles with respect to the prostate, which might reduce the efficacy of these techniques.

In this proof of principle study, we propose a novel IMRT adaptive planning method (previously patented by some of the authors of this article[23]), based on a library of plans, where the information provided by the IGRT system is used to adapt in real-time the treatment according to the current target position. Several studies in the literature have proposed the creation of patient-specific libraries of plans, mostly for bladder cancer RT. In these works, a plan-of-the-day is typically selected from the library to adapt to possible inter-fraction changes[24][25][26][27][28]. In the adaptive planning method introduced here, a combination of beams selected in real-time from different plans is delivered instead, in order to compensate for intra-fraction changes. The method proposed was implemented and evaluated dosimetrically against the standard PTV based planning approach (from now on referred to as “PTV planning method”) or against rigid isocenter shift.

## Material and methods

### Treatment simulation based on deformed CT

**Fig 1:**
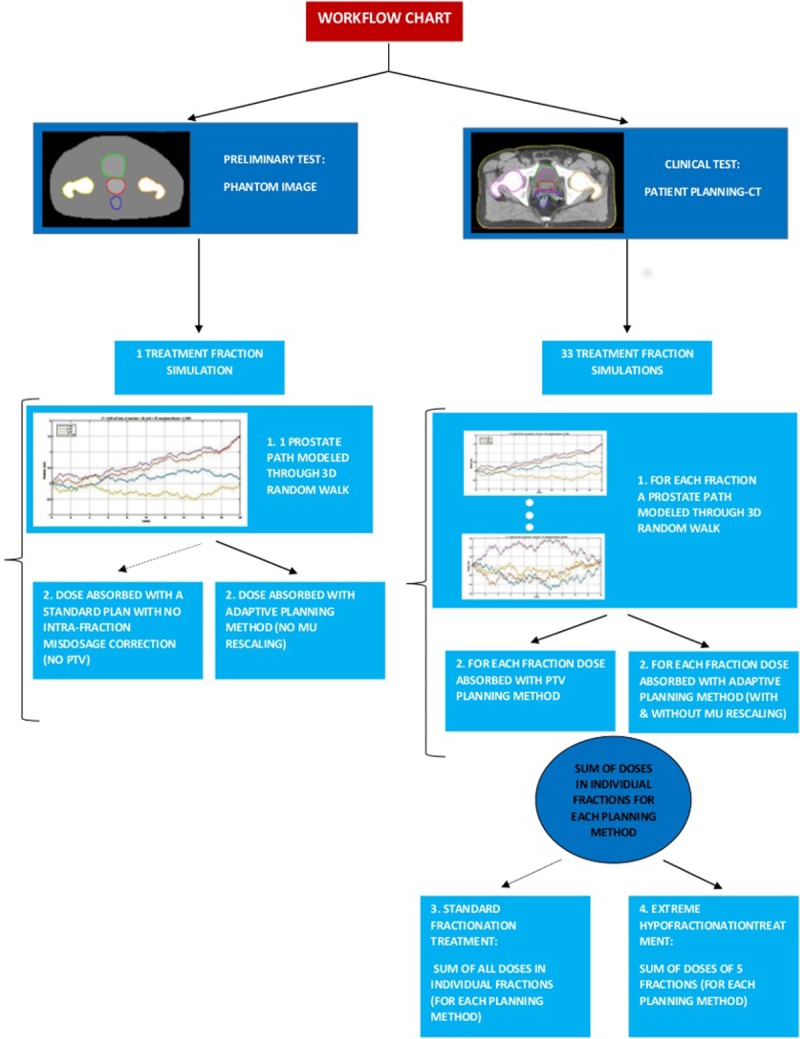
Workflow chart for the tests implemented.

A summary of the procedures implemented in this section is reported in the workflow chart in Fig 1.

### A. Treatment planning

#### A.1. Original plan on static treatment volume

Two standard IMRT treatment plans were created using a research version of the Philips Pinnacle^3^ treatment planning system (TPS) (version 16.0 Philips Radiation Oncology Systems, Fitchburg, WI, USA). The first plan was prepared on an in-silico phantom of the pelvic region, including the following regions of interest (ROIs) with the corresponding homogeneous density values: prostate, 1.09 g/cm^3^; bladder, 1.06 g/cm^3^; rectum, 1.03 g/cm^3^; and femurs, 2.14 g/cm^3^. The second plan was prepared on a computerized tomography (CT) image of an intermediate-risk prostate cancer (PCa) patient with a tumor locally extending to the seminal vesicles. The same ROIs as in the phantom plus the seminal vesicles were identified on the CT and contoured on the TPS by an experienced radiation oncologist (D.C.). The plans were optimized using five non pair-wise opposed photon beams (0, 30, 135, 225, 330 deg) and dosimetric objectives as suggested by RTOG [29] (Table 1).

**Table 1:** Dosimetric objectives on treatment volume and OARs for the whole treatment. The following conventions were used: Dv%>[d]Gy means that the dose that covered *v%* of the volume must be bigger than *d*Gy; V[d]Gy<[v]% means that the volume fraction that received at most *d*Gy must be smaller than *v%* of the volume.

		DOSIMETRIC OBJECTIVES
TREATMENT VOLUME	PTV\CTV a	D99% > 7600 cGy
	PTV\CTV a	D3% < 8300 cGy
	PTV\CTV a	D uniform = 8000 cGy
	SEMINAL VESICLES	D min > 5600 cGy
OAR	BLADDER	V5000 cGy < 60%
	BLADDER	V6500 cGy < 50%
	BLADDER	V6600 cGy < 40%
	BLADDER	V7000 cGy < 30%
	BLADDER	V7500 cGy < 25%
	BLADDER	V8000 cGy < 15%
	RECTUM	V6170 cGy < 50%
	RECTUM	V7429 cGy < 20%
	LEFT FEMUR	D max < 4000 cGy
	RIGHT FEMUR	D max < 4000 cGy

a: The dosimetric objective refers either to the PTV or to the CTV depending on the study case.

A dose of 2 Gy for 40 treatment fractions (hence 80 Gy overall [30]) was prescribed to the clinical target volume (CTV, corresponding to the prostate contour) for the phantom case and to a planning target volume (PTV), corresponding to an isotropic expansion of 5 mm[31] of the contoured CTV, for the patient case. The dose was computed using the Adaptive Convolve engine[32] with dose grid resolution of 0.4 cm. The optimization was performed with intensity modulation and no leaves segmentation to avoid connecting the work to a specific LINAC setup or model. The dose values obtained for the planning objectives are listed in Table 2 (see Results section). For simplicity, and without loss of generality, we considered a duration of 20 minutes for each treatment fraction, equally split in 4 minutes per beam.

#### A.2. Original plan on moving treatment volume

**A.2.1 Random walk model.** Intra-fraction prostate movement was simulated in MatLab (version 2014, The MathWorks, Inc., Natick, Massachusetts, United States) using a 3D random walk model[8][9]. At treatment start the treatment volume centre of mass was assumed to coincide with the prostate centre of mass (CM) in either the phantom image or the patient planning CT (computed from the segmented data). The walk steps were then iteratively created by randomly adding a fixed distance to the previous position, according to the variance reported in literature (see S1 Appendix). 600 prostate positions were sampled for each fraction. One path was calculated for the phantom case, producing a large shift at treatment end (1.3 cm). For the patient case, a total of 33 shifts, corresponding to as many fractions, were simulated. The resulting magnitude of prostate shifts at treatment end varied from less than 1 mm up to 1.5 cm. In about 49% of the cases simulated, the prostate end position was less than 0.5 cm distant from the original isocenter; in about 33% of the cases the distance was between 0.5 and 1 cm and in about 18% of the cases the drift was larger than 1 cm.

**A.2.2 Original image deformation.** Once the prostate motion simulation had been generated, the original image was modified to represent the different positions of the treatment volume corresponding to the different shifts. The procedure involved both rigid translation of the prostate and deformation of the surrounding tissue. Initially, a minimal spherical surface enclosing the whole prostate was determined and this surface was then expanded isotropically by 2 cm to define a “zero motion barrier” (ZMB). The region between the prostate and the ZMB spherical surface defined the region to be deformed, comprising most of bladder and rectum and the seminal vesicles. The region outside the ZMB remained the same as in the original images. The size of the ZMB was chosen such that it was large enough to ensure that the prostate would not cross it even for the largest shifts; and small enough so that it would not contain rigid structures, like the femurs, that should not be deformed.

Subsequently, a motion vector field (MVF) was determined describing the correspondence between the original positions of the voxel centres and the new positions of those centres after the shift. For the prostate, the voxels were rigidly shifted following the new position of the centre of mass. For the voxels between the surface of the prostate and the ZMB, the MVF was obtained through a 3D thin plate spline interpolation[33] between the prostate voxels and the stationary voxels outside the ZMB. An inverse motion vector field (IMVF) was then iteratively computed in MatLab[34].

To create the deformed image, a new grid with the same dimensions as the original image was defined. The IMVF was then used to create a correspondence between the voxels in the new grid and those in the original image. If a voxel in the new grid was mapped back to a point in the voxel centre of the original image, the intensity value of that voxel was the intensity of the voxel in the deformed grid. Otherwise the intensity in the deformed grid was calculated as trilinear interpolation of the adjacent voxels to the ‘off-grid’ point in the original image.

**A.2.3. Dose calculation.** An approximate dose distribution for each beam was computed by calculating the dose on either the deformed phantom image or the CT image corresponding to the average position of the prostate during that beam delivery. The prostate average position was computed according to the assumption already introduced that the dose delivery time was equally divided in 4 minutes per beam. So, for each treatment fraction, five deformed image volumes I_j_ (j = 1, …, 5) were generated. Each of the five deformed volumes was used as input to the corresponding original plan in the TPS and the dose during the corresponding j^th^ beam was calculated. Each dose volume was then mapped back to the reference image using the MVF associated with the deformed image. The total dose during the fraction was finally obtained by summing the dose from the five beams. Since the spatial relation between the voxels in the dose distribution and the position of the ROIs in the original image was known, it was possible to obtain the summed dose distribution and create the DVH for each ROI.

#### A.3. Adaptive plan on moving treatment volume

**A.3.1. Library of plans.** Two spheres with 5 mm and 10 mm radii respectively were created with the original prostate CM as centre. Then 20 quasi-equidistant points were sampled on each of the surfaces [35], in order to sample them as uniformly as possible, and these points were used as CMs of 40 virtual prostate volumes.

For each of these possible prostate CM positions, a new deformed image was generated with the same procedure as described in A.2.2. Finally, a new plan was optimized in the TPS for each of the points with the same characteristics and objectives as in the original plans (A.1.1), using as target the CTV for both the phantom and the patient CT, thus with no treatment margin applied. The plans were all created using a fast, fully automated algorithm that exploits the scripting capabilities of Pinnacle. The overall planning procedure took between 30 minutes and one hour.

**A.3.2. Prostate motion and plan selection.** The treatment delivery was simulated considering for each treatment fraction one of the prostate paths, whose generation was described in section A.2.1. After patient setup, the delivery of the plan created on the original image without PTV was initiated. Assuming that each beam lasts for 4 minutes, at the end of the delivery of the first beam the prostate CM and the corresponding tissue distribution at that moment were computed (according to the specific random walk selected). The plan optimized for the tissue distribution most similar to the current one was selected from the library of plans. Similarity was defined as the Euclidean distance between the current prostate CM and the prostate CM of the adaptive plan. The plan with the lowest Euclidean distance was selected and the second beam of that plan was delivered. The procedure was then repeated at the end of each beam until the end of the treatment.

**A.3.3. Dose calculation.** For each of the beams selected with the adaptive planning method, the dose distribution was calculated on the image volume corresponding to the average position of the prostate during that beam as described in section A.2.3, with the difference that now the beams in a treatment may come from different plans (selected as explained in section A.3.2). The dose for each ROI was then obtained following the procedure explained in section A.2.3.

**A.3.4. MU adaptation.** In the standard treatment planning phase, a MU value is assigned to each beam to optimize the dose distribution generated by that particular combination of beams composing the plan. Since in the proposed adaptive planning method the plan may consist of beams from different plans, it might be necessary to re-assign MU values based on the actual final plan delivered, in order to correct for possible discrepancies between the planned and the actually delivered dose. The MU re-computation was repeated at each beam end, even if no switch between plans had occurred. At each beam *m* end, the MU values (${MU}_{i}$) to be delivered for the subsequent beams were re-computed by multiplying by a positive scaling factor α each of the respective MU value (${MU}_{i,planning}$) previously obtained in the treatment planning phase:

$$MU_{i}=\alpha MU_{i,planning}$$

where i=m+1,…n, and *n* is the total number of beams.

The scaling factor α was calculated at each beam end by minimizing the objective function F, the same objective function employed in the Pinnacle TPS to generate the plans[36]. The estimated final dose *D*_*tot*,*m*_ for the treatment is the combination of the doses delivered by the past beams until *m* plus the summed dose from the beams from the plan selected for the following beam *m+1*:

$$\begin{array}{l} \alpha =\arg\;\min_{\alpha }(F(D_{tot,m}(\alpha )))\\ s.t.\;\alpha \geq 0\\ D_{tot,m}=\sum_{i=1}^{m}D_{i}+\alpha \sum_{j=m+1}^{n}D_{j} \end{array}$$

where n is the total number of beams. The dose predicted for the remaining beams was calculated assuming that the plan chosen for beam *m+1* was used until the end of the fraction. Since at that stage the prostate positions for the rest of the fraction were unknown, the assumption made for the remaining beams was that the prostate would stay in its current position. The doses computed for each beam were mapped on the original CT following the same procedure as described in A.3.3.

The function F was optimized in MatLab using a zero lower-bound constrained minimization[37], with a starting value for α of 1. Assuming that the MU values would remain reasonably similar to the ones generated in the planning procedure, the value of α was expected to be always around 1.

### Treatment simulation based on cone beam CT data

A Cone Beam CT of a PCa patient taken mid-treament to re-plan the treatment due to a significant shift of the target (~0.5 cm) was used to simulate intra-fraction tissue changes. Three approaches were tested: the standard PTV planning method, a rigid treatment isocenter shift to the new centroid of the target, and the adaptive planning method proposed in this work.

All the IMRT plans created in this part of the work were optimized using both the opening density matrices (ODM) and Direct Machine Parameter Optimization[38] (DMPO, Philips Radiation Oncology Systems, Fitchburg, WI, USA, which includes segmentation of the beams and control points creation) on an Elekta Synergy machine with Beam Modulator (Elekta AB, Stockholm, Sweden).

### B. Treatment planning

#### B.1. Original plan on static treatment volume

A treatment plan was prepared on the CT of an intermediate-risk PCa. The plan characteristics and objectives were kept as in the original plans (A.1.1), using as PTV a 0.5 cm uniform expansion of the CTV.

#### B.2. Original plan on moving treatment volume

A CBCT scan of the patient was used to simulate a possible intra-fraction change. As in section A.2.1., at treatment start the treatment volume CM was assumed to coincide with the CM in the planning CT. During the delivery of the first beam, the patient tissue distribution was assumed to be as in the planning CT. For the consecutive beams k (k = 2, …,5), the tissue configuration in the CBCT was instead considered. The dose computation for each of the beams was computed on the respective tissue distribution.

To compute the dose on the organ configuration of the CBCT, a new CT was generated (CT^CBCT^). The CBCT was rescaled to the same pixel dimension as the CT. The CT was then elastically deformed on the CBCT using the B-spline method in 3D Slicer (www.slicer.org). To obtain the ROIs for the new tissue distribution, the planning ROIs were propagated to the CT^CBCT^ using Pinnacle TPS and validated by a specialist. The computed doses were then summed as described in section A.2.3.

#### B.3. Rigid isocenter shift on moving treatment volume

A rigid isocenter shift was simulated on the treatment plan created as described in section B.1. The CT^CBCT^ and the respective ROIs were imported in the TPS. The PTV for the CTV in the CT^CBCT^ was created (with same characteristics as in B.1.) and the new isocenter position computed. The dose delivered by the beams k (k = 2,…,5) was updated and the dose delivered by the individual beams were summed as described in B.2.

#### B.4. Adaptive plan on moving treatment volume

The creation of the library of plans was performed as in section A.3.1. The plan selection was performed using the same procedure as in section A.3.2., selecting for the k^th^ beam delivery (k = 2,…,5) the plan optimized for the prostate with CM at shortest distance from the prostate CM in the CT^CBCT^. The dose computation procedure is described in section B.2.

### C. Dosimetric evaluation

The dosimetric comparison among the different planning strategies was performed using dose volume histograms (DVH) generated in MatLab. The DVH requirements used here were the same as used to create the plans (Table 1). For each treatment fraction, the dosimetric requirements were compared to the actual dose, in order to check in each case if the misdosage due to prostate motion happened and, in case it had, if the adaptive planning method would correct for it. Among the dosimetric objectives for the target, a uniformity condition on the CTV/PTV (depending on the planning procedure considered) was defined. The resulting deviation from uniformity was used as a qualitative measure for the presence of cold spots in those cases where the objectives on the prostate were not satisfied.

#### C.1. Treatment simulation based on deformed CT

For the phantom case, the comparison was performed between the original and the adaptive plans, both optimized using as target the CTVs, where the total dose was delivered in a single fraction.

For the clinical patient, two different treatment approaches were simulated:

1. IMRT treatment, 33 fractions for a total dose of 80 Gy (2.43 Gy per fraction).
2. IMRT treatment with extreme hypofractionation, 5 treatment fractions for a total dose of 80 Gy (16 Gy per fraction). Two separate cases were analysed:
  - 5 fractions randomly selected among the 33 in point 1. with a prostate final displacement at fraction end of 5 mm;
  - the 5 fractions, among the 33 in point 1. with prostate motion at fraction end larger than 1 cm, which, combined, generated the worst DVH in terms of target coverage.

MU rescaling (as described in section A.3.4) was applied to each of the simulated fractions.

#### C.2. Treatment simulation based on CBCT data

The comparison among the PTV planning method, the adaptive planning method and the rigid isocenter shift method was performed for a single treatment fraction, where the intra-fraction changes considered were based on the patient CBCT. The dosimetric comparison was performed considering the plans optimized using either pure fluence or DMPO.

## Results

### D. Treatment simulation based on deformed CT

#### D.1. Phantom case

**Fig 2:**
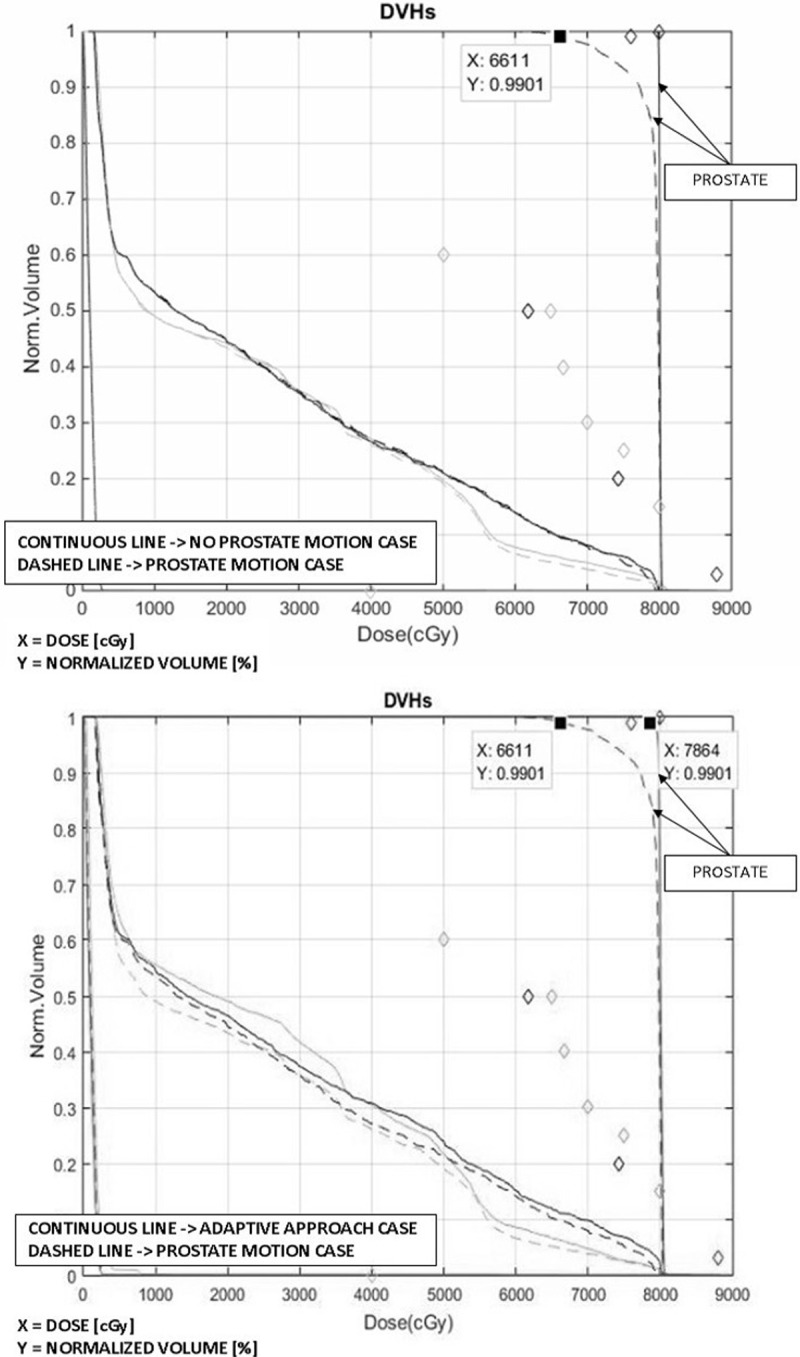
The DVHs for the plans generated for the phantom case. Top: PTV planning method with NO intra-fraction motion (continuous line); PTV planning method with intra-fraction motion (dashed line). Bottom: adaptive planning method (continuous line) and PTV planning method with intra-fraction motion (dashed line). The prostate shift at the end of the fraction is 1.25 cm. The arrows indicate the DVHs for the prostate (CTV). The DVHs are shown for all the organs involved: the diamonds correspond to the objectives imposed on each of the organs. The doses refer to the total treatment prescriptions.

For the phantom case, the results of the simulated fraction are presented in Fig 2. The DVH curves for the original plan delivered either to a static prostate (continuous line) or to a moving prostate during the fraction (dashed line) are shown in the top plot. In case of prostate motion, the dosimetric objective on 99% of the prostate volume (D99% > 7600 cGy) was not satisfied: the dose was 13% lower than required. For the adaptive planning method (without MU adaptation), instead, target coverage met the requirements (bottom graph in Fig 2). The dosimetric requirements on the OARs were satisfied in both cases.

#### D.2. Patient case

The dose values obtained for the adaptive planning method with and without MU rescaling were almost coincident (the difference was always a few cGy (see second and third columns in Table 2)), so the effect was considered negligible.

**Table 2:** Target and OAR doses for the volume fractions for which the requirements reported in Table 1 were imposed, for the standard IMRT treatment. Note: the dose values for the objective on the prostate (D**3**) and those for the femurs are not reported as the dosimetric objectives were always satisfied in our analysis.

	Dose [cGy]			
	PTV planning method	Adaptive planning method	Adaptive planning method, MU rescaled	PTV planning method NO motion
Prostate D99% > 7600 cGy	7894	7821	7826	7952
Seminal vesicles D min > 5600 cGy	5314	5843	5848	5594
Bladder V5000 cGy < 60%	4826	4997	4996	5184
Bladder V6500 cGy < 50%	5673	5569	5572	5942
Bladder V6600 cGy < 40%	6177	6145	6149	6595
Bladder V7000 cGy < 30%	6876	6790	6794	7205
Bladder V7500 cGy < 25%	7134	7133	7137	7580
Bladder V8000 cGy < 15%	7649	7669	7674	7908
Rectum V6170 cGy < 50%	6432	6144	6148	6382
Rectum V7429 cGy < 20%	7613	7465	7472	7632

In every single treatment fraction, when the adaptive planning method was applied all the dosimetric objectives on the treatment volume were always satisfied. Applying the PTV planning method, instead, several dosimetric requirements were not fulfilled for both prostate and seminal vesicles. In 5 fractions out of 33, the prostate was underdosed, possibly due to large shifts (1.25 cm, 1.04 cm, 1.30 cm, 1.12 cm and 1.19 cm respectively). For the seminal vesicles, relatively large underdosage was produced in almost every treatment fraction: in about 52% of the fractions, the underdosage was larger than 5% of the prescription dose.

**Fig 3:**
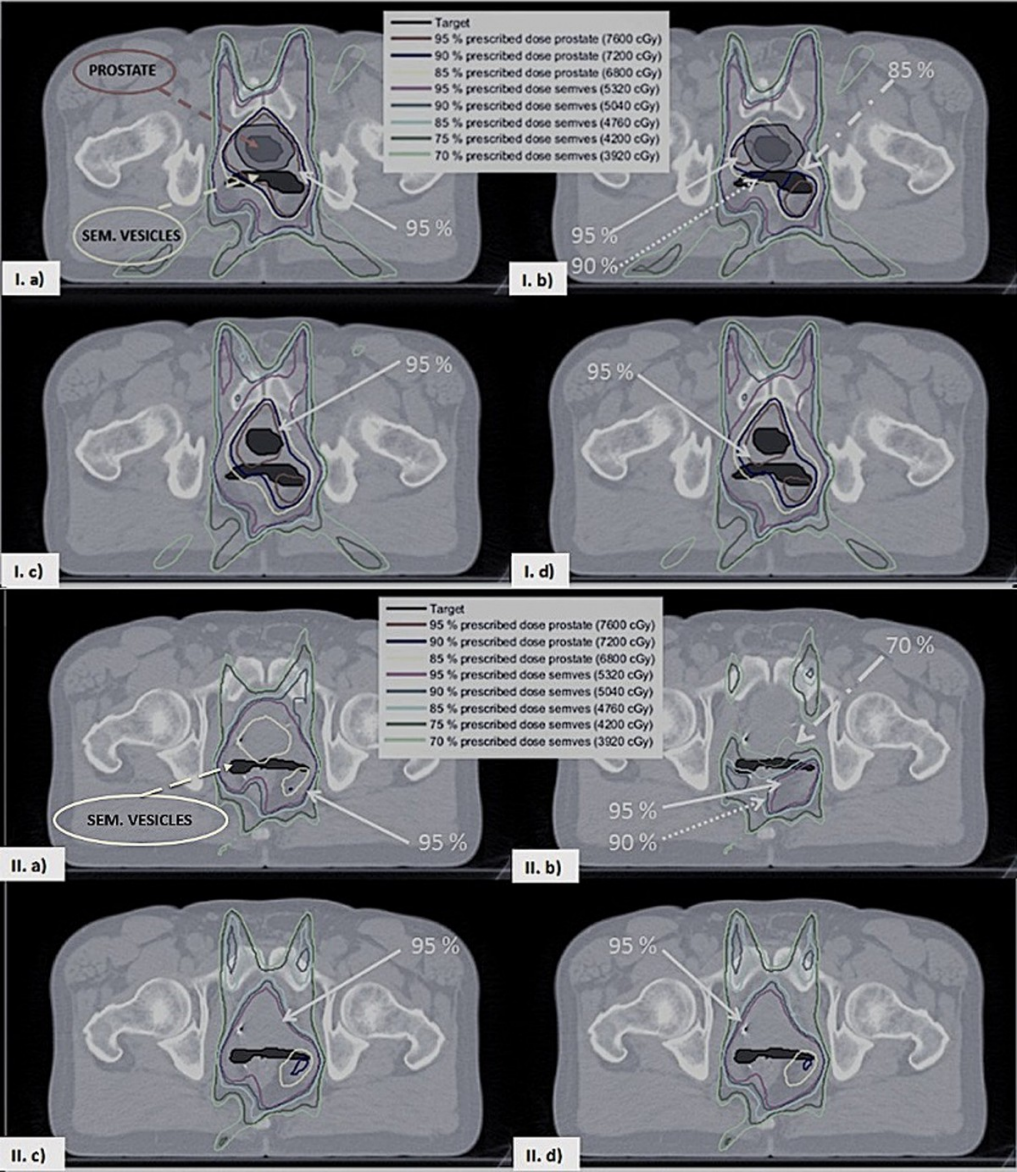
Isodose lines for two pelvic slices. In the top and the bottom figure a, b, c and d correspond respectively to the PTV planning method without/with intra-fraction motion and the adaptive planning method with/without MU rescaling (both with intra-fraction motion). In the top figure in a and b the PTV is shown as a grey shaded area surrounding the prostate. The prostate is visible only in the slice shown in the top figure.

For the fraction with the largest prostate misdosage (6% underdosage on 99% of the volume), isodose curves were inspected for each volume slice; the two axial projections with the largest cold spots (in the prostate and the seminal vesicles) are reported in Fig 3 (I and II respectively). For the PTV planning method, while without motion both the organs were absorbing the correct dose (Fig 3I and 3IIa), when the target motion was taken into account the prostate portion in the axial slice reported was completely missed by the 95% isodose line (Fig 3Ib). Also the part of the seminal vesicles in the axial slice in Fig 3Ib were mainly between the 70% and the 90% isodose lines. For the adaptive planning method, instead, prostate and seminal vesicles were treated correctly (Fig 3Ic and 3Id and 3IIc and 3IId).

Moreover, the adaptive planning method produced fewer and smaller dose deviations from the requirements on the OARs than the PTV planning method. The adaptive planning method reduced the number of fractions with misdosage by 50% for the bladder and by about 60% for the rectum. The frequency of severe misdosage (larger than 5%) was decreased by 96% for the rectum and by 73% for the bladder.

However, combining multiple fractions the misdosage produced was significantly reduced. In Tables 2 and 3 the results for the standard fractionation (33 fractions) and extreme hypofractionated IMRT (5 fractions) treatment, respectively, are shown. With the adaptive planning method, all the dosimetric objectives were satisfied for both treatment cases (Tables 2 and 3, column 2 and 3) and the doses on the OARs were always lower than the ones obtained with the PTV planning method. Moreover, with the PTV planning method, the seminal vesicles were underdosed by about 5% of the prescribed dose in the standard fractionation case (Table 2, column 1) and by about 4% of the prescribed dose in the extreme hypofractionated case (Table 3, column 1). The results related to the worst-case scenario, as described in point 2.b of section B, for the hypofractionated treatment are reported in S2 Appendix. Also in this case, applying the adaptive planning method there was a significant misdosage reduction.

**Table 3:** Target and OAR doses for the volume fractions for which the requirements reported in Table 1 were imposed, for the IMRT extreme hypofractionation treatment. Note: the dose values for the objective on the prostate (D**3**) and those for the femurs are not reported as the dosimetric objectives were always satisfied in our analysis.

	Dose [cGy]			
	PTV planning method	Adaptive planning method	Adaptive MU rescale planning method	PTV planning method NO motion
Prostate D99% > 7600 cGy	7919	7786	7711	7952
Seminal vesicles Dmin > 5600 cGy	5384	5848	5867	5594
Bladder V5000 cGy < 60%	4863	4990	5005	5184
Bladder V6500 cGy < 50%	5707	5574	5591	5942
Bladder V6600 cGy < 40%	6216	6139	6157	6595
Bladder V7000 cGy < 30%	6910	6804	6825	7205
Bladder V7500 cGy < 25%	7209	7148	7170	7580
Bladder V8000 cGy < 15%	7699	7676	7700	7908
Rectum V6170 cGy < 50%	6454	6124	6146	6382
Rectum V7429 cGy < 20%	7640	7415	7440	7632

### E. Treatment simulation based on CBCT data

**Fig 4:**
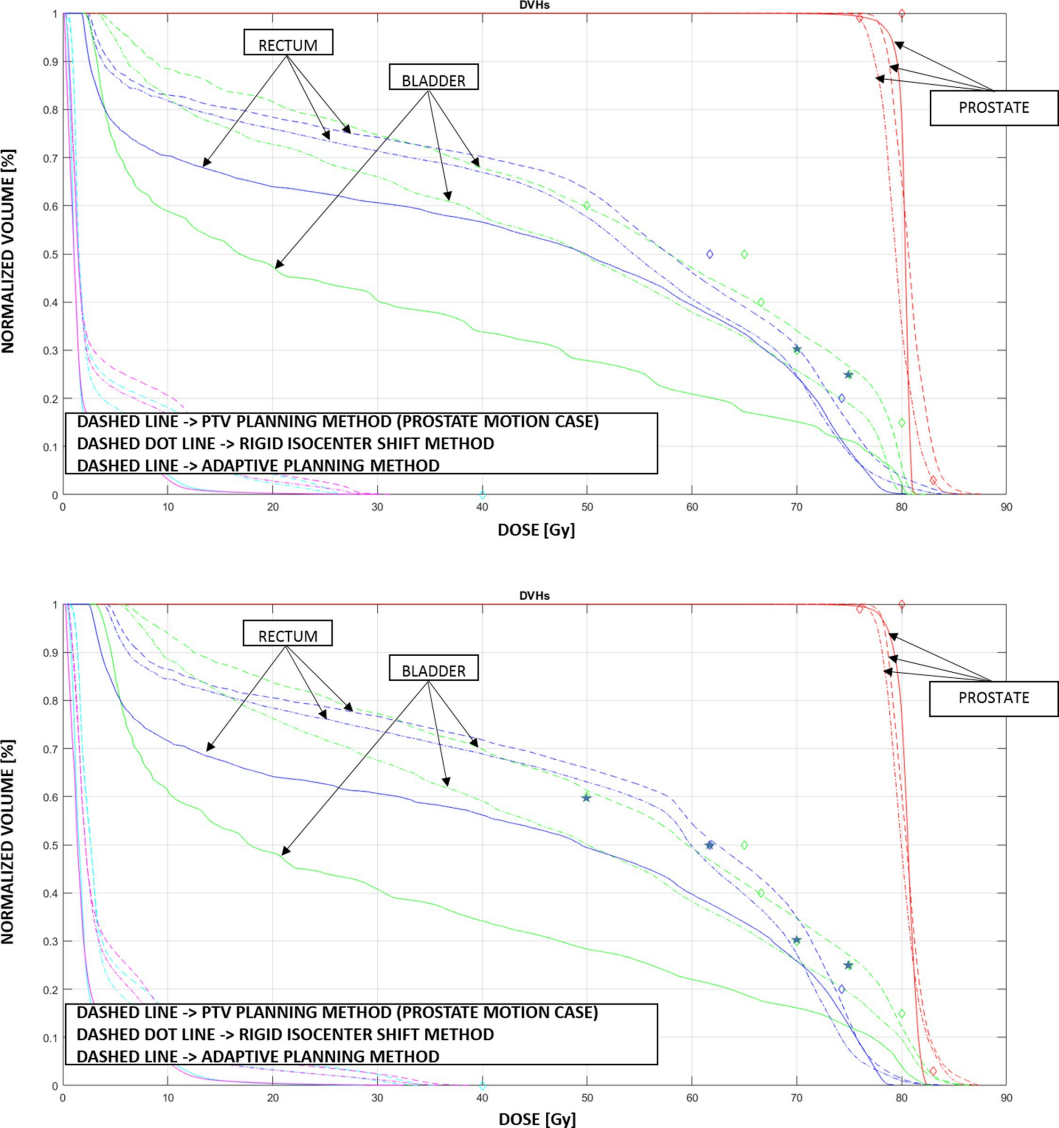
The DVHs for the plans optimized using pure fluence (top figure) and DMPO (bottom figure). PTV planning method (dashed line); PTV planning method with rigid isocenter shifts (dashed dot line); adaptive planning method (continuous line). The prostate shift at the end of the fraction is about 0.5 cm. The arrows indicate the DVHs for the prostate (CTV), bladder and rectum. The DVHs are shown for all the organs involved: the diamonds correspond to the objectives imposed on each of the organs and the stars correspond to the objectives not satisfied by the PTV planning method. The doses refer to the total treatment prescriptions.

The DVH curves for the PTV planning method, the rigid isocenter shift and the adaptive planning method of the simulated fraction are presented in Fig 4, using either pure fluence (top plot) or DMPO (bottom plot). The PTV planning approach (dashed line) with DMPO, when a shift of the target is simulated at the end of the first beam, produces always a poor uniformity in target coverage, one dosimetric objective failed for the rectum and three dosimetric objectives missed for the bladder, among which a 4.3% overdosage in the V7000 cGy < 30%. When pure fluence is used instead, only two dosimetric objectives are failed for the bladder and none for the rectum. The isocenter shift approach (dashed dot line) removes misdosage to OARs but still shows a poor target coverage. The adaptive planning method, instead, shows the best target coverage performance, and systematically the lowest dose to the OARs, with reductions up to 11% for the rectum and about 46% for the bladder, with respect to the standard PTV method, with a maximum dose decrease of 80% on the 60% bladder volume dosimetric objective.

## Discussion

In this proof of principle study, we compared an IMRT adaptive planning method with the standard IMRT planning method and with an isocenter shift correction approach. Although the concept of library of plans has already been introduced in the literature, the adaptive planning method proposed here is based on the selection of beams during each treatment fraction for intra-fraction adaptation, as opposed to the selection of the treatment of the day implemented in previous works[24][25][26][27][28]. The method produces inherently real-time adaptation, because no dose re-calculation or deformation is required at treatment, only a change of (pre-calculated) beams. Our hypothesis was that our method would be effective in a body region with a high degree of homogeneity in physical density: the pelvis for prostate EBRT. In such regions, in fact, small changes in tissue configurations will likely result in small changes (in terms of beam shapes and output values) in the treatment plans corresponding to the different anatomical configurations. This was indeed confirmed by the results we obtained, showing that beams can reasonably be combined from different plans because they have similar intensity matrices and similar MU values. This result is not trivial because, using inverse planning, the transition between beam shapes and MU values is not necessarily smooth when tissue configurations change.

For the standard fractionation study, after 33 fractions it was already clear that significant misdosage could happen, and that errors occurring in single fractions can compromise the entire treatment. The results are rescalable to any number of fractions, in any case, since all the plans were prepared for the total dose.

We assumed for simplicity that each beam would have a fixed duration of 4 minutes at a varying dose-rate. But it is absolutely equivalent (short of possible radiobiological effects of course) in terms of the results obtained here to consider standard fixed dose-rate beams with varying times. Furthermore, the doses estimated in this work are approximate doses, computed considering the average tissue distribution during the 4 minutes beam duration. A more precise dose computation could be performed by evaluating the dose for each prostate position simulated or reducing the time interval for which the dose is computed.

A major limitation for the clinical application of this method is the need of a real-time image-guidance system, which is currently not available in every hospital. We have also shown that our approach has advantages with respect to a simple intra-fraction isocenter shift, in particular in terms of dose to OARs. But it should be noted that its main strength is to compensate for deformation not rigid changes in position, which might be easily corrected for using a simple couch shift. Another drawback might be in the planning procedure, which could become time consuming due to the standard planning approval procedures, involving the formal clinical approval of both the DVH and the dose distribution before a plan can be delivered. In particular, the approval of the dose distribution is patient specific and hence there are no standards to determine which possible cold spots in the target, or hot spots for the OARs, can/cannot be accepted. Since for the adaptive planning method 40 plans are created, the dose distribution generated should be accepted for all of them, otherwise re-optimization would be necessary. Part of our future research plans are devoted to including in the automated procedure for the preparation of the plans a limitation on the region accessible by the prostate: the organ will not be allowed anymore to the whole isotropic volume around the original position but only to the volume physiologically reachable considering the possible movements of the gland (for example, the region where it would move due to the rotation around the apex). Contextually it would be interesting to implement in the deformation algorithm also the mechanical properties of the surrounding tissues to create ad-hoc potentially non-isotropic and non-linear deformation propagations. Potentially, it is foreseeable to implement in this workflow also automated approval procedures, based on assumptions of smooth propagation of the changes, which would request manual revision only of a few key plans in the library.

Further improvements could be applied to the adaptive planning method reducing the time before the adaptation time points, interchanging the control points (CPs) of different plans instead of the whole beams. We proved that changing beams is already sufficient to improve delivery accuracy, though; one of the next steps in our future work will be to investigate whether adding the significant complexity of dealing with intra-beam changes is clinically worthwhile. Apart from IMRT treatments, the adaptation using CPs could be applied also to VMAT[39][40] treatments. It is rather unlikely, though, that on the typically short treatment times of dynamic arc treatments the target will drift away enough to justify the correction proposed here. This would be the case however, when a larger number of beams are used, as it is clinical common practice for prostate IMRT treatments, where typically also 7 or 9 beams are used. Moreover, we believe this work has a fundamental clinical relevance as presently many more IMRT treatments are delivered than VMAT[41][42]. Worldwide VMAT is in fact not as widespread as it could be, due to licensing costs and need for new technology.

Another possible further refinement in the workflow could be implementing the dosimetric effect of rotation and deformation of the prostate. In the version presented here it has not been considered because several studies investigating intra-fraction prostate rotation/deformation showed that they are small compared to prostate rigid shifts and possibly not producing significant changes at a clinical level[43][44]. In particular, prostate deformations are rare and can reach a maximum of few millimetres[45][46][47]. For these reasons, adaptive planning techniques reported in the literature conventionally assume prostate rigid shifts as the only source of intra-fraction motion[48][49][50]. Many studies proved, though, that the seminal vesicles can experience considerable deformations as well and most of time move independently from the prostate[51][52][53][51][54]. While the average displacement of both organs increases with time, the displacement is larger for the seminal vesicles than for the prostate and can be on average larger than 1 cm in a time span of 15 min[54]. In line with these findings, in our study the relation between the prostate and the seminal vesicles was allowed to vary and therefore the seminal vesicles were not included in the GTV.

The weak correlation between prostate and motions of the seminal vesicles has significant implications for the PTV margin, especially in those cases where the seminal vesicles need to be treated. In line with Mutanga et al. [55], the 5 mm margin used for the PTV planning method simulated in this work was sufficient only to cover the prostate. Other studies proved in fact that to ensure dose coverage for both organs in 95.5% of the time a PTV margin of 10 mm should be considered; and to reach dose coverage in all the treatment fractions the PTV margin should be of 15 mm or even larger[56][57].

We proved in this proof-of-concept work that our adaptive planning strategy can produce full dosimetric target coverage in all cases considered for both the prostate and the seminal vesicles, even though no PTV margin was considered. In real clinical cases, though, a small PTV expansion should be considered to take into account all those uncertainties independent from prostate motion (e.g. contouring uncertainties). We do not expect, though, such a small margin to result in large dose increases, which would negatively affect the OARs. At the current stage, the dosimetric values on the OARs range from a few cGys up to a few Gys below the imposed dose limits, thus a small increase in dose will probably not affect the fulfilment of the constraints.

It is also important to notice that to actually deploy this adaptive technique for clinical treatments appropriate dose quality assurance (QA) guidelines should be adopted, incorporating time resolved measurements.

## Conclusions

The real-time adaptive planning method introduced in this proof-of-principle work for prostate cancer EBRT ensures, under the simulated treatment conditions, correct dose coverage for the treatment volume and significant toxicity reduction to the OARs. The adaptive planning method could be applied to standard fractionation treatments although in our study it proved most efficient when hypofrationation regimens are implemented. Further studies will be performed to test more extensively the proposed adaptive planning method and to evaluate it at a full clinical level.

## Supporting information

S1 Appendix Appendix A. (PDF)

S2 Appendix Appendix B. (PDF)
