# Supplementary material for: Real-time adaptive planning method for radiotherapy treatment delivery for prostate cancer patients, based on a library of plans accounting for possible anatomy configuration changes
Source: PLoS One. 2019 Feb 28;14(2):e0213002. doi: 10.1371/journal.pone.0213002 (PMC6394960; doi:10.1371/journal.pone.0213002)
Supplement: S1 Appendix — (PDF) [file pone.0213002.s001.pdf]

Appendix A

According to Ballhausen et al., intra-fraction prostate motion can be modelled as a 3D random walk [1,2]. In 1D, the random walk can be represented as:

$$x_n = x_{n-1} \pm \mathit{\Delta}$$

where x_n is the position after step n for n ≥1, $\mathit{\Delta}$ is the step size and the sign is picked randomly with equal probability. For this model, as the number of steps increases the expectation of the displacement is zero, since a negative or a positive step is equally likely. Thus, the random walk can be better characterized using the average of the square of the displacement or variance:

$\sigma_x^2 \equiv \langle (x_n - x_0)^2 \rangle = n\Delta^2$ where x_0 is the initial position. We assumed the random walk characteristics of the prostate motion were the same in all directions, so the average squared displacement in 3D was:

$$\sigma^2 \equiv \langle (\vec{r}_n - \vec{r}_0)^2 \rangle = 3n\Delta^2$$

where $\vec{r}_n = (x_n, y_n, z_n)$ is the position in 3D after n steps.

The number of steps in a random walk is equal to the duration T of the random walk divided by the time resolution δ_t.

$$n = \frac{T}{\delta_t}$$

So we can write:

$$\sigma^2 = 3b^2T$$

where $b^2 \equiv \Delta^2/\delta_t$.

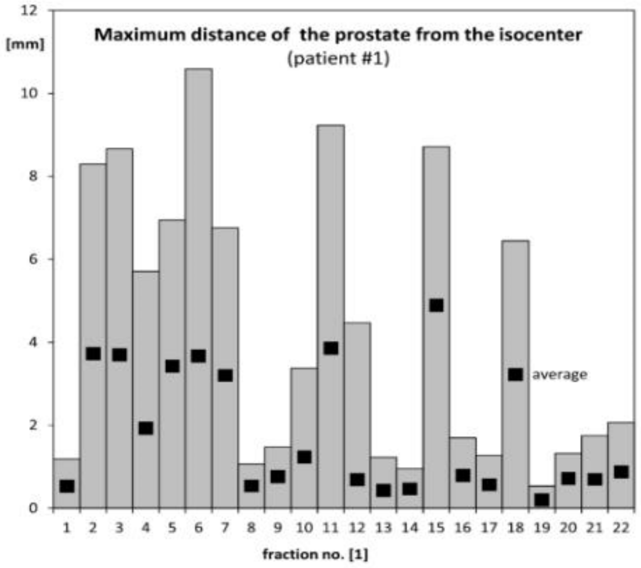

Figure 1: The maximum and the average prostate displacement (black squares) for a patient during different treatment fractions [2].

To compute b^2, we used the prostate displacements reported in Ballhausen's work (Figure 1) [2]. In the figure the maximum and the average displacement for the prostate in different fractions are reported for a prostate cancer patient. The ensemble average of the square of the distance travelled by the prostate at time t ($\langle\sqrt{\langle r^2\rangle_t}\rangle$) was extracted directly from the graph shown in Figure 1 [2]. The relation between the average displacement and the variance in time can be expressed as

$$\langle\sqrt{\langle r^2\rangle_t}\rangle \equiv \frac{1}{T}\int_0^T \sqrt{3b^2 t}\ dt = \frac{2\sqrt{3}}{3} b\sqrt{T}$$

where T is the treatment time duration (20 min). Thus

$$b^2 = \frac{3}{4}\frac{\langle\sqrt{\langle r^2\rangle_t}\rangle^2}{T}$$

Using the values of b^2 determined from the 22 values obtained from Figure 1, different random walks were generated in MatLab (version 2014, The MathWorks, Inc., Natick, Massachusetts, United States). The step size Δ was obtained by choosing a time resolution δ_t of 2 seconds. Among the 22 values used, the variance generating the largest displacement was selected:

$$b^2 = 0.0090\ cm^2/min$$

$$\Delta = \sqrt{b^2\delta_t} = 0.017\ cm$$

The reason for selecting a variance which generated a large drift of the prostate was to have a high probability that some of the random walks in our work would have a large final displacement (about 1 cm or more). This way, some critical cases could be included in the simulations.

References:

1. H Ballhausen, M Li, N-S Hegemann UG and CB. Intra-fraction motion of the prostate is a random walk. 2014;549. doi:10.1088/0031-9155/60/2/549.

2. Ballhausen H, Reiner M, Kantz S, Belka C, Söhn M. The random walk model of intrafraction movement. *Phys Med Biol*. 2013;58(7):2413-2427. doi:10.1088/0031-9155/58/7/2413.
