# Supplementary material for: Real-time adaptive planning method for radiotherapy treatment delivery for prostate cancer patients, based on a library of plans accounting for possible anatomy configuration changes
Source: PLoS One. 2019 Feb 28;14(2):e0213002. doi: 10.1371/journal.pone.0213002 (PMC6394960; doi:10.1371/journal.pone.0213002)
Supplement: S2 Appendix — (PDF) [file pone.0213002.s002.pdf]

Appendix B

Table 4: IG-IMRT hypofractionation treatment representing the worst scenario analysis case. Target and OAR doses for the volume fractions for which the requirements reported in Table 1 were imposed, for this treatment case. Note: the dose values for the objective on the prostate (D_3) and those for the femurs are not reported as the dosimetric objectives were always satisfied in our analysis.

	Dose values corresponding to dosimetric requirements			
	**PTV planning method**	**Adaptive planning method**	**Adaptive MU rescale planning method**	**PTV planning method NO motion**
Prostate $D_{99\%}$ > 7600 cGy	7476	7758	7757	7952
Seminal vesicles D_{min} > 5600 cGy	4468	5943	5939	5594
Bladder $V_{5000\ cGy}$ < 60%	3946	4895	4887	5184
Bladder $V_{6500\ cGy}$ < 50%	4863	5371	5370	5942
Bladder $V_{6600\ cGy}$ < 40%	5614	6031	6032	6595
Bladder $V_{7000\ cGy}$ < 30%	6034	6640	6642	7205
Bladder $V_{7500\ cGy}$ < 25%	6381	6953	6953	7580
Bladder $V_{8000\ cGy}$ < 15%	7041	7509	7509	7908
Rectum $V_{6170\ cGy}$ < 50%	6698	6281	6278	6382
Rectum $V_{7429\ cGy}$ < 20%	7764	7592	7592	7632
